# Supplementary material for: Health Impacts of the COVID-19 Lockdown Measure in a Low Socio-Economic Setting: A Cross-Sectional Study on Reunion Island
Source: Int J Environ Res Public Health. 2022 Oct 26;19(21):13932. doi: 10.3390/ijerph192113932 (PMC9657094; doi:10.3390/ijerph192113932)
Supplement: Supplementary file 1 [file ijerph-19-13932-s001.zip › Supplementary-Materials-R2.pdf]

Table S1. Factors associated with a high level of stress in the adult population during the COVID-19 lockdown (Ré-Conf-ISS cross-sectional study: Reunion Island, May 13 and July 22, 2020)—first nested multivariable regression models (M1, M2, and M3).

Factors	**M1** [1]		**M2** [1]		**M3** [1]	
	**aOR (95% CI)**	***P***	**aOR (95% CI)**	***P***	**aOR (95% CI)**	***P***
**Housing conditions during the lockdown:**						
Daily annoyances in the home (Yes vs. No)	-	-	-	-	1.13 (0.74-1.73)	0.569
**SARS-CoV-2 epidemic exposure:**						
Knowledge of an infected close relative [2] (Yes vs. No/don't know)	-	-	-	-	1.36 (0.72-2.56)	0.338
**Individual socio-economic position:**						
Experience of financial difficulties [3] (Yes vs. No/don't know)	-	-	1.58 (1.04-2.41)	0.033	1.59 (1.05-2.41)	0.029
**Demographic factors and baseline health state:**						
Sex (Man vs. Woman)	0.46 (0.31-0.69)	<0.001	0.47 (0.32-0.71)	<0.001	0.48 (0.32-0.72)	<0.001
Age (60–74 yrs vs. 18–59 yrs)	0.44 (0.27-0.73)	0.002	0.47 (0.29-0.78)	0.003	0.48 (0.29-0.78)	0.003
Age (75–90 yrs vs. 18–59 yrs)	0.70 (0.39-1.28)	0.247	0.80 (0.45-1.43)	0.450	0.83 (0.46-1.49)	0.525
History of chronic disease [4] (Yes vs. No/don't know)	1.66 (1.15-2.40)	0.007	1.63 (1.13-2.35)	0.009	1.62 (1.13-2.34)	0.010

A high level of stress during the lockdown was a binary endpoint defined by a score >6 on a scale ranging from 0 (no stress) to 10 (maximum imaginable stress) [15]. The reference category was "No high level of stress" (score ≤6). [1] A first multivariable binary logistic regression model (M1) included sex, age and history of chronic disease. We then included an indicator of individual socio-economic position in a second multivariable model (M2). In M3, we added an indicator of housing conditions during the lockdown. M3 also included an indicator of epidemic indirect exposure potentially degrading psychological state: the knowledge of a SARS-CoV-2 infected close relative living on Reunion Island or elsewhere. All multivariable models (M1, M2, and M3) were computed on available data provided same sample size (n = 880). [2] Living on Reunion Island or elsewhere. [3] Related to the COVID-19 health crisis. [4] Health problem requiring regular visits to the doctor. aOR: adjusted odds ratio. 95% CI: 95% confidence interval. *P*: *P*-value. vs.: versus.

Table S2. Factors associated with a deteriorated psychological state in the adult population during the COVID-19 lockdown (Ré-Conf-ISS cross-sectional study: Reunion Island, May 13 and July 22, 2020)—first nested multivariable regression models (M1, M2, and M3).

Factors	M1 [1] aOR (95% CI)	M1 [1] *P*	M2 [1] aOR (95% CI)	M2 [1] *P*	M3 [1] aOR (95% CI)	M3 [1] *P*
**Housing conditions during the lockdown:**						
Living in overcrowded conditions (Yes vs. No)	-	-	-	-	1.12 (0.79-1.57)	0.525
**SARS-CoV-2 epidemic exposure:**						
Knowledge of an infected close relative [2] (Yes vs. No/don't know)	-	-	-	-	1.33 (0.63-2.80)	0.459
**Individual socio-economic position:**						
Experience of financial difficulties [3] (Yes vs. No/don't know)	-	-	2.07 (1.50-2.87)	<0.001	2.08 (1.50-2.89)	<0.001
**Demographic factors and baseline health state:**						
Sex (Man vs. Woman)	0.81 (0.63-1.04)	0.103	0.84 (0.65-1.09)	0.201	0.85 (0.65-1.10)	0.217
Age (60–74 yrs vs. 18–59 yrs)	0.48 (0.33-0.71)	<0.001	0.52 (0.35-0.78)	0.002	0.54 (0.35-0.83)	0.005
Age (75–90 yrs vs. 18–59 yrs)	0.53 (0.31-0.92)	0.023	0.63 (0.36-1.10)	0.106	0.66 (0.38-1.16)	0.151
History of chronic disease [4] (Yes vs. No/don't know)	1.37 (0.99-1.89)	0.052	1.34 (0.97-1.86)	0.075	1.35 (0.98-1.86)	0.068

A deteriorated psychological state during the lockdown was a binary endpoint defined by the presence of at least one of the following five situations: currently not feeling calm in daily life and/or lockdown-related worsening of a psychological condition pre-existing the epidemic and/or a psychological condition triggered by the epidemic or lockdown and/or perception of one's social relationships as very bad or somewhat bad and/or worries about ones' job or work situation in the immediate future. The reference category is "No deteriorated psychological state". [1] A first multivariable binary logistic regression model (M1) included sex, age and history of chronic disease. We then included an indicator of individual socio-economic position in a second multivariable model (M2). In M3, we added an indicator of housing conditions during the lockdown. M3 also included an indicator of epidemic indirect exposure potentially degrading psychological state: the knowledge of a SARS-CoV-2 infected close relative living on Reunion Island or elsewhere. All multivariable models (M1, M2, and M3) were computed on available data provided same sample size (n = 852). [2] Living on Reunion Island or elsewhere. [3] Related to the COVID-19 health crisis. [4] Health problem requiring regular visits to the doctor. aOR: adjusted odds ratio. 95% CI: 95% confidence interval. *P: P*-value. vs.: versus.

Table S3. Factors associated with lockdown positive appreciation in the adult population during the COVID-19 lockdown (Ré-Conf-ISS cross-sectional study: Reunion Island, May 13 and July 22, 2020)—first nested multivariable regression models (M1, M2, and M3—to be continued).

	M1 [1]				M2 [1]			
	Level of engagement in relaxing activities		No positive appreciation or don't know		Level of engagement in relaxing activities		No positive appreciation or don't know	
Factors	aIRR (95% CI)	*P*	aOR (95% CI)	*P*	aIRR (95% CI)	*P*	aOR (95% CI)	*P*
**Housing conditions during the lockdown:**								
A potentially high mental load (Yes vs. No)	-	-	-	-	-	-	-	-
Optimal access to the outside (Yes vs. No)	-	-	-	-	-	-	-	-
**Individual socio-economic position:**								
University degree as education level (Yes vs. No)	-	-	-	-	1.11 (1.05-1.18)	<0.001	0.74 (0.53-1.04)	0.083
Housing occupancy status (Homeowners vs. Rent-free)	-	-	-	-	1.02 (0.99-1.06)	0.222	0.95 (0.73-1.24)	0.727
Housing occupancy status (Renters vs. Rent-free)	-	-	-	-	0.95 (0.92-0.98)	0.005	1.02 (0.79-1.30)	0.904
**Demographic factors and baseline health state:**								
Sex (Man vs. Woman)	0.99 (0.93-1.05)	0.643	1.60 (1.21-2.13)	0.001	0.98 (0.93-1.04)	0.470	1.60 (1.20-2.13)	0.001
Age (40–59 yrs vs. 18–39 yrs)	0.99 (0.95-1.04)	0.706	0.78 (0.63-0.98)	0.031	1.00 (0.96-1.04)	0.938	0.79 (0.63-0.99)	0.042
Age (60–74 yrs vs. 18–39 yrs)	0.95 (0.90-1.01)	0.108	1.10 (0.83-1.46)	0.515	0.95 (0.90-1.01)	0.121	1.10 (0.81-1.50)	0.541
Age (75–90 yrs vs. 18–39 yrs)	1.01 (0.95-1.09)	0.696	1.97 (1.30-3.00)	0.002	1.00 (0.94-1.07)	0.944	1.93 (1.27-2.94)	0.002
History of chronic disease [3] (Yes vs. No/don't know)	0.98 (0.92-1.04)	0.434	1.12 (0.86-1.47)	0.400	0.98 (0.92-1.04)	0.443	1.11 (0.84-1.45)	0.461

Table S3. (End).

	M3 [1]			
	Level of engagement in relaxing activities		No positive appreciation or don't know	
**Factors**	**aIRR (95% CI)**	***P***	**aOR (95% CI)**	***P***
**Housing conditions during the lockdown:**				
A potentially high mental load (Yes vs. No)	1.01 (0.96-1.07)	0.664	0.70 (0.50-0.98)	0.036
Optimal access to the outside (Yes vs. No)	1.10 (1.03-1.17)	0.003	0.92 (0.68-1.26)	0.608
**Individual socio-economic position:**				
University degree as education level (Yes vs. No)	1.11 (1.05-1.18)	0.001	0.75 (0.53-1.06)	0.105
Housing occupancy status (Homeowners vs. Rent-free)	1.02 (0.98-1.06)	0.357	0.96 (0.74-1.25)	0.762
Housing occupancy status (Renters vs. Rent-free)	0.96 (0.93-0.99)	0.009	1.05 (0.81-1.35)	0.721
**Demographic factors and baseline health state:**				
Sex (Man vs. Woman)	0.98 (0.92-1.03)	0.379	1.56 (1.17-2.08)	0.002
Age (40–59 yrs vs. 18–39 yrs)	0.99 (0.96-1.03)	0.770	0.82 (0.65-1.03)	0.085
Age (60–74 yrs vs. 18–39 yrs)	0.96 (0.91-1.02)	0.168	1.04 (0.77-1.42)	0.783
Age (75–90 yrs vs. 18–39 yrs)	1.01 (0.95-1.07)	0.826	1.83 (1.19-2.80)	0.006
History of chronic disease [2] (Yes vs. No/don't know)	0.98 (0.92-1.03)	0.397	1.09 (0.83-1.44)	0.523

The lockdown positive appreciation score was a count endpoint defined as the sum of behaviors or attitudes for having engaged in at least one of the following thirteen relaxing activities: refocusing, enjoying the quiet, breathing less polluted air, having conversations, reading, writing, watching movies/series, playing games, cooking, DIY, gardening, doing nothing, and doing something else that feels good. The higher the score was, the better was positive appreciation of the lockdown. A zero (minimum) value meant no positive appreciation of the lockdown or don't know. [1] A first multivariable regression model (M1) included sex, age and history of chronic disease. We then included indicators of individual socio-economic position in a second multivariable model (M2). In M3, we added indicators of housing conditions during the lockdown. All multivariable models (M1, M2, and M3) were computed on available data provided same sample size (n = 850). [2] Health problem requiring regular visits to the doctor. Figures are from multivariable zero-inflated negative binomial (ZINB) regression models. In the zero-inflated part of the ZINB regression model, the reference category was "A positive appreciation of the lockdown for at least one of the thirteen relaxing activities". aIRR: adjusted incidence rate ratio. aOR: adjusted odds ratio. 95% CI: 95% confidence interval. *P*: *P*-value. vs.: versus.

.

Table S4. Factors associated with an increase in at least one addictive behavior in the adult population during the COVID-19 lockdown (Ré-Conf-ISS cross-sectional study: Reunion Island, May 13 and July 22, 2020)—first nested multivariable regression models (M1, M2, and M3).

	**M1** [1]		**M2** [1]		**M3** [1]	
**Factors**	**aOR (95% CI)**	***P***	**aOR (95% CI)**	***P***	**aOR (95% CI)**	***P***
**Housing conditions during the lockdown:**						
Poor Internet access (No vs. Yes)	-	-	-	-	1.21 (0.89-1.63)	0.221
**Individual socio-economic position:**						
University degree as education level (Yes vs. No)	-	-	2.04 (1.38-3.02)	<0.001	2.00 (1.36-2.96)	<0.001
Housing occupancy status (Renters vs. Homeowners)	-	-	1.80 (1.28-2.54)	<0.001	1.79 (1.26-2.55)	0.001
Housing occupancy status (Rent-free vs. Homeowners)	-	-	1.18 (0.82-1.72)	0.372	1.20 (0.83-1.73)	0.342
**Demographic factors and baseline health state:**						
Sex (Man vs. Woman)	0.91 (0.68-1.21)	0.497	0.87 (0.66-1.16)	0.343	0.87 (0.65-1.15)	0.319
Age (40–59 yrs vs. 18–39 yrs)	0.84 (0.60-1.19)	0.326	0.96 (0.67-1.38)	0.838	0.96 (0.67-1.37)	0.820
Age (60–74 yrs vs. 18–39 yrs)	0.38 (0.26-0.55)	<0.001	0.49 (0.34-0.72)	<0.001	0.49 (0.34-0.72)	<0.001
Age (75–90 yrs vs. 18–39 yrs)	0.33 (0.18-0.59)	<0.001	0.44 (0.24-0.81)	0.009	0.46 (0.25-0.85)	0.012
History of chronic disease [2] (Yes vs. No/don't know)	0.78 (0.56-1.07)	0.127	0.80 (0.57-1.11)	0.177	0.80 (0.57-1.11)	0.180

An increase in addictive behaviors during the lockdown was a binary endpoint. It concerned the use of at least one of the following items: screens, alcohol, tobacco (cigarette/e-cigarette), or psychotropic drugs (natural or synthetic cannabinoid-based drugs), and participation in virtual happy hours. The reference category was "No increase in addictive behavior during the lockdown". [1] A first multivariable binary logistic regression model (M1) included sex, age and history of chronic disease. We then included indicators of individual socio-economic position in a second multivariable model (M2). In M3, we added an indicator of housing conditions during the lockdown. All multivariable models (M1, M2, and M3) were computed on available data provided same sample size (n = 870). [2] Health problem requiring regular visits to the doctor. aOR: adjusted odds ratio. 95% CI: 95% confidence interval. *P*: *P*-value. vs.: versus.

Table S5. Factors associated with difficulties in accessing food in the adult population during the COVID-19 lockdown (Ré-Conf-ISS cross-sectional study: Reunion Island, May 13 and July 22, 2020)—first nested multivariable regression models (M1, M2, and M3—to be continued).

	**M1** [1]				**M2** [1]			
	**Number of difficulties**		**No difficulty**		**Number of difficulties**		**No difficulty**	
**Factors**	**aIRR (95% CI)**	***P***	**aOR (95% CI)**	***P***	**aIRR (95% CI)**	***P***	**aOR (95% CI)**	***P***
**Housing conditions during the lockdown:**								
A potentially high mental load (Yes vs. No)	-	-	-	-	-	-	-	-
Living in overcrowded conditions (Yes vs. No)	-	-	-	-	-	-	-	-
**Individual socio-economic position:**								
Usually living alone (Yes/No)	-	-	-	-	0.84 (0.71-0.99)	0.052	1.74 (1.03-2.95)	0.038
Experience of financial difficulties [3] (Yes vs. No/don't know)	-	-	-	-	1.24 (1.10-1.40)	<0.001	1.03 (0.67-1.57)	0.909
**Demographic factors and baseline health state:**								
Sex (Man vs. Woman)	0.91 (0.79-1.04)	0.156	1.10 (0.71-1.68)	0.674	0.93 (0.82-1.06)	0.296	1.11 (0.73-1.70)	0.623
Age (40–59 yrs vs. 18–39 yrs)	1.16 (1.03-1.32)	0.017	0.69 (0.49-0.97)	0.031	1.14 (1.01-1.28)	0.038	0.72 (0.51-1.02)	0.066
Age (60–74 yrs vs. 18–39 yrs)	1.04 (0.88-1.23)	0.634	1.25 (0.87-1.78)	0.222	1.05 (0.89-1.25)	0.537	1.19 (0.82-1.73)	0.366
Age (75–90 yrs vs. 18–39 yrs)	0.80 (0.59-1.07)	0.125	3.09 (1.72-5.56)	<0.001	0.82 (0.62-1.09)	0.166	2.89 (1.59-5.27)	0.001
History of chronic disease [4] (Yes vs. No/don't know)	0.98 (0.87-1.10)	0.686	1.01 (0.70-1.46)	0.951	0.96 (0.85-1.08)	0.477	1.01 (0.69-1.48)	0.957

Table S5. (End).

Factors	M3 [1]			
	Number of difficulties		No difficulty	
	aIRR (95% CI)	*P*	aOR (95% CI)	*P*
**Housing conditions during the lockdown:**				
A potentially high mental load (Yes vs. No)	1.05 (0.92-1.21)	0.459	0.53 (0.30-0.95)	0.034
Living in overcrowded conditions (Yes vs. No)	1.13 (0.99-1.29)	0.075	1.48 (0.88-2.50)	0.143
**Individual socio-economic position:**				
Usually living alone (Yes/No)	0.87 (0.73-1.04)	0.122	1.71 (0.99-2.96)	0.055
Experience of financial difficulties [2] (Yes vs. No/don't know)	1.21 (1.06-1.37)	0.004	0.99 (0.62-1.58)	0.969
**Demographic factors and baseline health state:**				
Sex (Man vs. Woman)	0.94 (0.83-1.07)	0.369	1.08 (0.70-1.68)	0.732
Age (40–59 yrs vs. 18–39 yrs)	1.11 (0.98-1.26)	0.088	0.73 (0.50-1.06)	0.096
Age (60–74 yrs vs. 18–39 yrs)	1.08 (0.91-1.28)	0.392	1.15 (0.78-1.70)	0.479
Age (75–90 yrs vs. 18–39 yrs)	0.85 (0.64-1.14)	0.272	2.87 (1.57-5.22)	0.001
History of chronic disease [3] (Yes vs. No/don't know)	0.97 (0.86-1.09)	0.593	1.01 (0.68-1.49)	0.974

The score of difficulties in accessing food during the lockdown was a count endpoint defined as the sum of difficulties for the following items: fresh fruits and vegetables, garlic/onions, eggs, flour, and other essential food items. The higher the score was, the higher was the number of difficulties. A zero (minimum) value meant no difficulty. [1] A first multivariable regression model (M1) included sex, age and history of chronic disease. We then included indicators of individual socio-economic position in a second multivariable model (M2). In M3, we added indicators of housing conditions during the lockdown. All multivariable models (M1, M2, and M3) were computed on available data provided the same sample size (n = 851). [2] Related to the COVID-19 health crisis. [3] Health problem requiring regular visits to the doctor. Figures are from multivariable zero-inflated negative binomial (ZINB) regression models. In the zero-inflated part of the ZINB regression model, the reference category was "≥ 1 difficulty in accessing food during the lockdown". aIRR: adjusted incidence rate ratio. aOR: adjusted odds ratio. 95% CI: 95% confidence interval. *P: P*-value. vs.: versus.

Table S6. Factors associated with a decrease in physical activity in the adult population during the COVID-19 lockdown (Ré-Conf-ISS cross-sectional study: Reunion Island, May 13 and July 22, 2020)—first nested multivariable regression models (M1, M2, and M3).

	**M1** [1]		**M2** [1]		**M3** [1]	
**Factors**	**aOR (95% CI)**	***P***	**aOR (95% CI)**	***P***	**aOR (95% CI)**	***P***
**Housing conditions during the lockdown:**						
Poor Internet access (No vs. Yes)	-	-	-	-	1.23 (0.92-1.66)	0.167
Optimal access to the outside (Yes vs. No)	-	-	-	-	1.35 (0.97-1.87)	0.076
**Individual socio-economic position:**						
University degree as education level (Yes vs. No)	-	-	1.66 (1.18-2.33)	0.004	1.59 (1.13-2.25)	0.009
**Demographic factors and baseline health state:**						
Sex (Man vs. Woman)	1.00 (0.75-1.32)	0.989	1.00 (0.75-1.32)	0.989	0.99 (0.74-1.31)	0.930
Age (40–59 yrs vs. 18–39 yrs)	0.99 (0.70-1.42)	0.972	1.02 (0.72-1.45)	0.923	1.02 (0.72-1.45)	0.908
Age (60–74 yrs vs. 18–39 yrs)	1.26 (0.85-1.87)	0.258	1.33 (0.89-1.99)	0.161	1.36 (0.90-2.04)	0.140
Age (75–90 yrs vs. 18–39 yrs)	0.43 (0.25-0.75)	0.003	0.47 (0.27-0.82)	0.008	0.51 (0.29-0.90)	0.020
History of chronic disease [2] (Yes vs. No/don't know)	0.86 (0.63-1.19)	0.370	0.88 (0.64-1.22)	0.444	0.88 (0.64-1.21)	0.437

A decrease in physical activity during the lockdown was a binary endpoint defined by the presence of at least one of the following three situations: sports practice at a club or an association before the lockdown and/or use of a neighborhood sports facility before the lockdown and/or perceived decrease in physical activity during the lockdown. The reference category was "No increase in physical activity during the lockdown". [1] A first multivariable binary logistic regression model (M1) included sex, age, and history of chronic disease. We then included an indicator of individual socio-economic position in a second multivariable model (M2). In M3, we added indicators of housing conditions during the lockdown. All multivariable models (M1, M2, and M3) were computed on available data provided same sample size (n = 865). [2] Health problem requiring regular visits to the doctor. aOR: adjusted odds ratio. 95% CI: 95% confidence interval. *P: P*-value. vs.: versus.

Table S7. Factors associated with a delayed medical appointment at the request of the medical secretariat in the adult population during the COVID-19 lockdown (Ré-Conf-ISS cross-sectional study: Reunion Island, May 13 and July 22, 2020)—first nested multivariable regression models (M1, M2, and M3).

	M1 [1]		M2 [1]		M3 [1]	
**Factors**	**aOR (95% CI)**	***P***	**aOR (95% CI)**	***P***	**aOR (95% CI)**	***P***
**Housing conditions during the lockdown:**						
A potentially high mental load (Yes vs. No)	-	-	-	-	1.35 (0.86-2.14)	0.195
Optimal access to the outside (Yes vs. No)	-	-	-	-	0.84 (0.56-1.28)	0.428
Daily annoyances in the home (Yes vs. No)	-	-	-	-	1.47 (0.95-2.28)	0.084
**Individual socio-economic position:**						
University degree as education level (Yes vs. No)	-	-	1.56 (1.06-2.28)	0.023	1.52 (1.04-2.23)	0.031
Complementary health insurance status (Positive vs. Negative) [2]	-	-	1.50 (0.99-2.26)	0.051	1.62 (1.05-2.52)	0.030
**Demographic factors and baseline health state:**						
Sex (Man vs. Woman)	0.70 (0.50-0.97)	0.034	0.68 (0.48-0.95)	0.022	0.72 (0.51-1.02)	0.064
Age (40–90 yrs vs. 18–39 yrs)	0.69 (0.45-1.05)	0.085	0.68 (0.44-1.05)	0.081	0.70 (0.46-1.08)	0.106
History of chronic disease [3] (Yes vs. No/don't know)	2.05 (1.35-3.12)	<0.001	2.18 (1.42-3.37)	<0.001	2.24 (1.44-3.47)	<0.001

A medical appointment during the lockdown delayed at the request of the medical secretariat was a binary endpoint. The reference category was "No delayed medical appointment at all or medical appointment delayed for another reason (than at the medical secretariat and on its own initiative)". [1] A first multivariable binary logistic regression model (M1) included sex, age and history of chronic disease. We then included indicators of individual socio-economic position in a second multivariable model (M2). In M3, we added indicators of housing conditions during the lockdown. All multivariable models (M1, M2, and M3) were computed on available data provided same sample size (n = 740). [2] Positive: benefiting from a private complementary health insurance. Negative: lacking complementary health insurance or benefiting from the free public complementary health insurance. [3] Health problem requiring regular visits to the doctor. aOR: adjusted odds ratio. 95% CI: 95% confidence interval. *P*: *P*-value. vs.: versus.

Table S8. Factors associated with a delayed medical appointment on its own initiative in the adult population during the COVID-19 lockdown (Ré-Conf-ISS cross-sectional study: Reunion Island, May 13 and July 22, 2020)—first nested multivariable regression models (M1, M2, and M3).

	**M1** [1]		**M2** [1]		**M3** [1]	
**Factors**	**aOR (95% CI)**	***P***	**aOR (95% CI)**	***P***	**aOR (95% CI)**	***P***
**Housing conditions during the lockdown:**						
A potentially high mental load (Yes vs. No)	-	-	-	-	1.56 (0.97-2.51)	0.068
Optimal access to the outside (Yes vs. No)	-	-	-	-	1.67 (1.06-2.63)	0.026
Daily annoyances in the home (Yes vs. No)	-	-	-	-	1.70 (1.07-2.71)	0.024
**Individual socio-economic position:**						
University degree as education level (Yes vs. No)	-	-	1.06 (0.66-1.73)	0.799	0.99 (0.60-1.64)	0.978
Complementary health insurance status (Positive vs. Negative) [2]	-	-	1.06 (0.71-1.58)	0.780	1.13 (0.75-1.70)	0.570
**Demographic factors and baseline health state:**						
Sex (Man vs. Woman)	0.55 (0.37-0.80)	0.002	0.54 (0.37-0.80)	0.002	0.57 (0.38-0.84)	0.005
Age (40–90 yrs vs. 18–39 yrs)	0.85 (0.54-1.33)	0.466	0.84 (0.54-1.32)	0.453	0.93 (0.58-1.49)	0.759
History of chronic disease [3] (Yes vs. No/don't know)	1.18 (0.78-1.80)	0.425	1.19 (0.79-1.81)	0.405	1.24 (0.82-1.89)	0.308

A medical appointment during the lockdown delayed on its own initiative was a binary endpoint. The reference category was "No delayed medical appointment at all or medical appointment delayed for another reason (than on its own initiative and at the request of the medical secretariat)". [1] A first multivariable binary logistic regression model (M1) included sex, age and history of chronic disease. We then included indicators of individual socio-economic position in a second multivariable model (M2). In M3, we added indicators of housing conditions during the lockdown. All multivariable models (M1, M2, and M3) were computed on available data provided same sample size (n = 697). [2] Positive: benefiting from a private complementary health insurance. Negative: lacking complementary health insurance or benefiting from the free public complementary health insurance. [3] Health problem requiring regular visits to the doctor. aOR: adjusted odds ratio. 95% CI: 95% confidence interval. *P*: *P*-value. vs.: versus.

Table S9. Factors independently associated with a delayed medical appointment on its own initiative in the adult population during the COVID-19 lockdown (Ré-Conf-ISS cross-sectional study: Reunion Island, May 13 and July 22, 2020)—fully adjusted multivariable models (M4).

	M4			
	Daily annoyances in the home:			
	**Yes** (n = 132)		**No** (n= 537)	
**Factors**	**aOR (95% CI)**	***P***	**aOR (95% CI)**	***P***
**Increase in large neighborhood deprivation level** (continuous)	1.60 (1.06-2.43)	0.025	0.84 (0.71-0.99)	0.040
**Housing conditions during the lockdown:**				
A potentially high mental load (Yes vs. No)	1.67 (0.75-3.72)	0.208	1.48 (0.83-2.64)	0.187
Optimal access to the outside (Yes vs. No)	3.12 (1.06-9.18)	0.039	1.33 (0.82-2.16)	0.241
Daily annoyances in the home (Yes vs. No)	-	-	-	-
**Individual socio-economic position:**				
University degree as education level (Yes vs. No)	1.27 (0.31-5.19)	0.741	0.88 (0.53-1.44)	0.611
Complementary health insurance status (Positive vs. Negative) [1]	0.37 (0.15-0.90)	0.029	1.45 (0.88-2.38)	0.145
**Demographic factors and baseline health state:**				
Sex (Man vs. Woman)	1.68 (0.78-3.62)	0.185	0.38 (0.23-0.61)	<0.001
Age (40–90 yrs vs. 18–39 yrs)	0.63 (0.30-1.32)	0.225	1.03 (0.57-1.87)	0.910
History of chronic disease [2] (Yes vs. No/don’t know)	2.03 (0.83-4.96)	0.123	1.00 (0.65-1.53)	0.995

A medical appointment during the lockdown delayed on its own initiative was a binary endpoint. The reference category was “No delayed medical appointment at all or medical appointment delayed for another reason (than on its own initiative and at the request of the medical secretariat)”. [1] Negative: lacking complementary health insurance or benefiting from the free public complementary health insurance. Positive: benefiting from a private complementary health insurance. [2] Health problem requiring regular visits to the doctor. Figures are from a multivariable binary logistic regression models adjusted on large neighborhood deprivation level, housing conditions, individual socio-economic position, demographic factors, baseline health state, and stratified by daily annoyances in the home during the lockdown (Yes/No). aOR: adjusted odds ratio. 95% CI: 95% confidence interval. *P*: *P*-value. vs.: versus.

Table S10. Factors associated with economic or psychological violence against women during the COVID-19 lockdown (Ré-Conf-ISS cross-sectional study: Reunion Island, May 13 and July 22, 2020)—first nested multivariable regression models (M1, M2, and M3).

Factors	M1 [1] aOR (95% CI)	M1 [1] *P*	M2 [1] aOR (95% CI)	M2 [1] *P*	M3 [1] aOR (95% CI)	M3 [1] *P*
**Housing conditions during the lockdown:**						
Daily annoyances in the home (Yes vs. No)	-	-	-	-	2.38 (1.06-5.32)	0.035
A potentially high mental load (Yes vs. No)	-	-	-	-	3.33 (1.38-8.03)	0.007
**Individual socio-economic position:**						
Experience of financial difficulties [2] (Yes vs. No/don't know)	-	-	1.40 (0.61-3.22)	0.428	1.03 (0.45-2.39)	0.939
Socio-professional category (Unemployed vs. Employed)	-	-	2.58 (1.01-6.58)	0.048	3.02 (1.05-8.71)	0.041
Socio-professional category (Retirees vs. Employed)	-	-	0.21 (0.04-1.13)	0.069	0.31 (0.04-2.24)	0.248
Housing occupancy status (Renters vs. Homeowners)	-	-	2.50 (1.01-6.18)	0.048	1.59 (0.63-3.96)	0.324
Housing occupancy status (Rent-free vs. Homeowners)	-	-	1.98 (0.65-6.00)	0.228	1.70 (0.53-5.43)	0.371
**Baseline health state:**						
History of chronic disease [3] (Yes vs. No/don't know)	2.74 (1.36-5.53)	0.005	4.16 (1.91-9.07)	<0.001	5.24 (2.26-12.14)	<0.001

Violence against women during the lockdown was a binary endpoint defined by nine situations of economic or psychological abuse linked to the lockdown and the COVID-19 pandemic. The reference category was "No violence suffered during the lockdown". [1] A first multivariable binary logistic regression model (M1) included history of chronic disease. We then included indicators of individual socio-economic position in a second multivariable model (M2). In M3, we added indicators of housing conditions during the lockdown. All multivariable models (M1, M2, and M3) were computed on available data provided same sample size (n = 558). [2] Related to the COVID-19 health crisis. [3] or health problem requiring regular visits to the doctor. aOR: adjusted odds ratio. 95% CI: 95% confidence interval. *P*: *P*-value. vs.: versus.

Table S11. Factors associated with the number of health problems in children (< 18 yrs) during the COVID-19 lockdown (Ré-Conf-ISS cross-sectional study: Reunion Island, May 13 and July 22, 2020)—first nested multivariable regression models (M1, M2, and M3).

Factors	M1 [1] aIRR (95% CI)	M1 [1] *P*	M2 [1] aIRR (95% CI)	M2 [1] *P*	M3 [1] aIRR (95% CI)	M3 [1] *P*
**Housing conditions during the lockdown:**						
Daily annoyances in the home (Yes vs. No)	-	-	-	-	1.19 (0.94-1.50)	0.148
**Individual socio-economic position:**						
Parent usually living alone (Yes/No)	-	-	1.59 (0.99-2.54)	0.053	1.56 (0.99-2.45)	0.056
Experience of financial difficulties [2] (Yes vs. No/don't know)	-	-	1.46 (1.17-1.81)	0.001	1.41 (1.14-1.76)	0.002
**Demographic factors:**						
Sex of parent (Man vs. Woman)	0.75 (0.56-0.99)	0.047	0.76 (0.58-0.99)	0.045	0.77 (0.59-0.99)	0.050
Age of parent (40–90 yrs vs. 18–39 yrs)	0.94 (0.75-1.18)	0.583	0.95 (0.77-1.18)	0.652	0.95 (0.77-1.17)	0.650

Health problems in children during the lockdown concerned the entire sibling group and consisted of the following: worry, anxiety, stress, sleep and eating disorders, concentration or attention problems, and learning difficulties. They were identified based on the statements by 354 parents confined with their child(ren) during the lockdown and summarized in a sum score. The higher the score was, the lower was the health of children. [1] A first multivariable regression model (M1) included sex and age of parent. We then included indicators of parental socio-economic position in a second multivariable model (M2). In M3, we added an indicator of housing conditions during the lockdown. All multivariable models (M1, M2, and M3) were computed on available data provided same sample size (n = 346). Figures are from multivariable negative binomial regression models with an offset [=log(size of entire sibling group)] to adjust the at-risk population size. [2] Related to the COVID-19 health crisis. aIRR: adjusted incidence rate ratio. 95% CI: 95% confidence interval. *P*: *P*-value. vs.: versus.

Table S12. Factors associated with at least one health problem in children (< 18 yrs) during the COVID-19 lockdown (Ré-Conf-ISS cross-sectional study: Reunion Island, May 13 and July 22, 2020)—first nested multivariable regression models (M1, M2, and M3).

	**M1** [1]		**M2** [1]		**M3** [1]	
**Factors**	**aOR (95% CI)**	***P***	**aOR (95% CI)**	***P***	**aOR (95% CI)**	***P***
**Housing conditions during the lockdown:**						
Daily annoyances in the home (Yes vs. No)	-	-	-	-	2.05 (1.12-3.75)	0.020
**Individual socio-economic position:**						
Parent usually living alone (Yes/No)	-	-	1.86 (0.44-7.82)	0.398	1.59 (0.38-6.66)	0.529
Experience of financial difficulties [2] (Yes vs. No/don't know)	-	-	1.56 (0.93-2.62)	0.090	1.45 (0.87-2.41)	0.152
**Demographic factors:**						
Sex of parent (Man vs. Woman)	0.56 (0.35-0.89)	0.015	0.57 (0.36-0.91)	0.019	0.60 (0.37-0.95)	0.031
Age of parent (40–90 yrs vs. 18–39 yrs)	0.69 (0.44-1.07)	0.099	0.71 (0.46-1.10)	0.127	0.71 (0.46-1.11)	0.131

Health problems in children during the lockdown concerned the entire sibling group and consisted of the following: worry, anxiety, stress, sleep and eating disorders, concentration or attention problems, and learning difficulties. They were identified based on the statements by 354 parents confined with their child(ren) during the lockdown and summarized in a sum score. The higher the score was, the lower was the health of children. At least one health problem in children was a binary endpoint based on the health children score ≥ 1 (Yes/No). The reference category was "No health problem in children during the lockdown". [1] A first multivariable binary logistic regression model (M1) included sex and age of parent. We then included indicators of parental socio-economic position in a second multivariable model (M2). In M3, we added an indicator of housing conditions during the lockdown. All multivariable models (M1, M2, and M3) were computed on available data provided same sample size (n = 352). [2] Related to the COVID-19 health crisis. aOR: adjusted odds ratio. 95% CI: 95% confidence interval. *P: P*-value. vs.: versus.
