# Supplementary material for: Health Impacts of the COVID-19 Lockdown Measure in a Low Socio-Economic Setting: A Cross-Sectional Study on Reunion Island
Source: Int J Environ Res Public Health. 2022 Oct 26;19(21):13932. doi: 10.3390/ijerph192113932 (PMC9657094; doi:10.3390/ijerph192113932)
Supplement: Supplementary file 1 [file ijerph-19-13932-s001.zip › Suppl-Materials-Study-Questionnaire.pdf]

Réunion, Confinement, Inégalités Sociales de Santé :

Ré-Conf-ISS

Enquête téléphonique à la sortie du confinement

Cahier d'observation

- Version du 18/05/2020 –

Numéro d'enregistrement du participant :

|__|__|__| - |__|__|__|

Numéro d'enregistrement = Code grand quartier (001 à 114) - numéro séquentiel incrémentable (001 à 999)

Promoteur

Centre Hospitalier Universitaire de la Réunion

CHU Sud Réunion

BP350 97748 St-Pierre Cedex

Investigateur principal

FIANU Adrian

CHU Sud Réunion, CIC 1410

adrian.fianu@chu-reunion.fr

Consignes pour remplir le cahier d'observation de l'étude Ré-Conf-ISS

1. Le cahier d'observation est imprimé sur du papier simple.

2. N'utilisez pas d'abréviation mais écrivez en toutes lettres.

3. Complétez en MAJUSCULES de façon lisible les items à données libres.

4. Complétez le numéro d'enregistrement sur l'en-tête de chaque page.

5. Inscrivez un seul caractère par case.

6. Valeurs numériques :
   - cadrez les valeurs numériques à droite
   - n'ajoutez pas de virgules
   - ne laissez pas de cases vides, mettez un zéro devant si nécessaire.

   Incorrect |_2_|_1_|__| Correct |_0_|_2_|_1_|

7. Lorsque la réponse doit être reportée dans des cases fermées, cochez la case correspondante :

   Par exemple : Oui ☐ Non ☒

8. Dates : indiquez les dates au format demandé : jour-mois-année (jj/mm/aaaa)

9. En cas d'absence de données, barrez les cases vides et utilisez les codes suivants :

   NA : non applicable ou sans objet
   NC : non connu
   NSP : ne sait pas

10. Chaque erreur doit être barrée d'un trait (la valeur erronée doit rester lisible), corrigée à côté, datée et paraphée (avec les initiales du correcteur) avec un stylo à bille rouge. N'utilisez pas de gommettes ou « blanco » pour effacer les erreurs. Mais laisser les visibles.

Ré-Conf-ISS	NUMERO D'ENREGISTREMENT : |__|__|__| - |__|__|__|

Date d'appel :	[_J_] [_J_] /[_M_] [_M_]/[_2_] [_0_] [_2_] [_0_]

Nom et prénom de l' enquêteur/trice :	____

Information sur l'étude *(texte à lire)* :

« L'objectif de cette enquête téléphonique **anonyme**, conçue par le Centre d'Investigation Clinique de la Réunion, est de décrire, à la fin du confinement, l'état de santé de la population réunionnaise selon ses caractéristiques socio-économiques. Cette étude devrait permettre de mieux connaitre les populations vulnérables en situation de confinement, à la Réunion. Elle servira aussi à proposer des ajustements et des mesures d'accompagnement pour prévenir les difficultés liées au confinement à la Réunion. »

Avez-vous déjà été contacté pour cette enquête ?	☐ Oui ☐ Non<br>***Si Oui : fin de l'enquête.***

1) Vérification des critères d'éligibilité et renvoi vers la page Web de l'étude

Texte à lire : « Les trois prochaines questions portent sur votre lieu de résidence pendant le confinement (du 17 mars au 11 mai) : »

1.1. Dans quelle commune de l'île avez-vous été confiné ?	________________________
1.2 Quel était le code postal ?	|_9_|_7_|_4_|__|__|
1.3. Dans quel quartier avez-vous été confiné ? (En clair + code)<br>*Voir la 1ere colonne du tableau dans le document intitulé* ***Enquête téléphonique Ré-Conf-ISS : Procédure d'échantillonnage***	________________________<br>|__|__|__|
1.4. Habitiez-vous déjà à la Réunion avant le début du confinement (avant le 17 mars 2020)	☐ Oui ☐ Non<br>***Si Non : fin de l'enquête.***
1.5. Avez-vous vécu la totalité de l'épisode du confinement à la Réunion (8 semaines)	☐ Oui ☐ Non<br>***Si Non : fin de l'enquête.***
1.6. Etes-vous sous curatelle ?	☐ Oui ☐ Non<br>***Si Oui : fin de l'enquête.***
1.7. Quel est votre âge ?	|__|__|__| ans<br>***Si < 18 ans : fin de l'enquête.***
1.8. Avant de commencer, êtes-vous d'accord pour participer à cette étude ?	☐ Oui ☐ Non<br>***Si Non : fin de l'enquête.***

Texte à lire et à compléter avec le numéro d'enregistrement du/de la participant(e) :

« Si vous souhaitez plus d'information sur l'étude ou finalement vous opposer à l'utilisation de vos données personnelles pour cette étude, vous pouvez vous rendre sur la page internet du CHU de la Réunion (https://www.chu-reunion.fr) et faire une recherche avec le mot « enquête », puis sélectionner « Réunion, Confinement, Inégalités Sociales de Santé : **Ré-Conf-ISS** une enquête lancée par le CIC* et le CHU ».

Attention, le rappel de votre numéro d'enregistrement (qui est **|__|__|__| - |__|__|__|**) est nécessaire pour toute démarche de prise de contact avec les chercheurs de l'étude. »

2) Caractéristiques socio-économiques individuelles

2.1. Est-ce que vous êtes :	☐ une femme ☐ un homme ☐ autre genre

<u>Pour les femmes uniquement</u> (texte à lire) :

« Cette enquête va aborder sur la fin du questionnaire des questions sur les violences subies pendant le confinement. Vous sentez-vous en toute sécurité (seule) pour répondre à ces questions ?

Si ce n'est pas le cas, nous pouvons convenir d'un rendez-vous téléphonique, pendant lequel ces conditions seront réunies, pour réaliser l'enquête : »

Date du RDV : |__|__| /|__|__| /2020

Heure du RDV : |__|__|H : |__|__|Min

2.2. Habituellement vivez-vous seul(e) ?	☐ Oui ☐ Non
2.3. Quel est le diplôme le plus élevé que vous ayez obtenu :	☐ Aucun diplôme<br>☐ Certificat de formation générale (CFG), certificat d'études primaires, diplôme national du brevet (BEPC ou brevet des collèges)<br>☐ CAP ou BEP<br>☐ Baccalauréat ou diplôme équivalent<br>☐ Diplôme de l'enseignement supérieur<br>☐ Autre à préciser en clair (attestations, etc.) :<br>________________________________

2.4. Quelle est votre catégorie socioprofessionnelle ?<br><br>(*1 seul choix possible. Si un doute persiste sur la CSP alors noter en clair*)	☐ Agriculteur exploitant ou conjointe sur exploitation<br><br>☐ Artisan, commerçant, chef d'entreprise ou conjoint/e collaboratrice/teur<br><br>☐ Cadre, profession intellectuelle supérieure (ingénieur, médecin…)<br><br>☐ Profession intermédiaire (professeur des écoles, infirmière, assistante sociale, technicien, contremaître, agent de maîtrise…)<br><br>☐ Employé (employée de bureau ou de commerce, garde d'enfants, agent de service…)<br><br>☐ Ouvrier<br><br>☐ Retraité *(puis aller à la question 2.9.)*<br><br>☐ Parent au foyer *(puis aller à la question 2.9.)*<br><br>☐ Etudiant *(puis aller à la question 2.9.)*<br><br>☐ Au chômage *(puis aller à la question 2.9.)*<br><br>☐ Autre sans activité professionnelle, à préciser en clair : ____<br>*(puis aller à la question 2.9.)*
2.5. Si vous travaillez, quel emploi occupez-vous (en clair) ?	____
2.6. Votre emploi est-il à risque d'exposition à l'épidémie de Covid-19 ?<br><br>(c'est-à-dire : personnels soignant, travailleurs sociaux, transporteurs, hôtes de caisses et employés de commerces, facteurs, chargés de collecte des déchets, forces de l'ordre, pompiers, gardiens d'immeubles, entre autres métiers à risque)	☐ Oui ☐ Non ☐ Sans objet

2.7. Depuis le Covid-19, vos conditions de travail :	☐ Sont les mêmes qu'avant<br>☐ Plus souvent sur mon lieu de travail<br>☐ Moins souvent sur mon lieu de travail<br>☐ En télétravail<br>☐ En arrêt de travail<br>☐ Au chômage partiel<br>☐ Travail perdu<br>☐ Sans objet (pas d'activité professionnelle)<br>☐ Autre situation à préciser en clair : ________________
2.8. Est-ce que votre rémunération a été maintenue suite à l'épidémie de Covid-19 ?	☐ Oui ☐ Non ☐ Je ne sais pas encore
2.9. Avez-vous ressenti des difficultés financières liées à la crise du Covid-19 ?	☐ Oui ☐ Non ☐ NSP

3) Logement, équipement et environnement proche de l'habitat occupé pendant le confinement

Texte à lire :

« Les questions qui suivent se rapportent toutes à la période du confinement (du 17 mars au 11 mai 2020). Si vous avez occupé plusieurs lieux de confinement, merci de répondre pour celui occupé le plus longtemps sur la période. »

3.1. Votre lieu de confinement était :	☐ Une maison ou une kaz a ter<br><br>☐ Un appartement dans une résidence privée<br><br>☐ Un appartement d'un bailleur social (SHLMR, SIDR, ..)<br><br>☐ Un logement collectif institutionnel (EHPAD, foyers pour publics vulnérables)<br><br>☐ Un logement précaire (véhicule, tente, etc.)<br><br>☐ Sans logement (dans la rue)<br><br>☐ Autre situation à préciser en clair :<br>____
3.2. Dans ce logement y avait-il les problèmes suivants :<br><br>- Trop d'habitants<br>- Problème d'humidité<br>- Problème de bruit<br>- Problème de température (trop chaud / trop froid)	 <br><br>☐ Oui ☐ Non<br>☐ Oui ☐ Non<br>☐ Oui ☐ Non<br>☐ Oui ☐ Non
3.3. Dans ce logement, vous étiez :	☐ Propriétaire<br><br>☐ Locataire<br><br>☐ Co-locataire<br><br>☐ Hébergé(e) à titre gracieux

3.4. Combien de personnes habitaient dans ce logement y compris-vous :	|__|__| *recueillir le nombre exact (≥ 1)*
3.5. Et combien d’enfants mineurs dans ce logement (vos enfants, ceux du voisins hébergés gracieusement, tous les enfants) :<br>- De 0-3 ans :<br>- De 4-10 ans :<br>- De 11-14 ans :<br>- De 15-18 ans :	 |__|__| *recueillir le nombre exact (≥0)*<br>|__|__| *recueillir le nombre exact (≥0)*<br>|__|__| *recueillir le nombre exact (≥0)*<br>|__|__| *recueillir le nombre exact (≥0)*
3.6. Combien y avait-il de chambres dans ce logement :	|__|__| *recueillir le nombre exact de chambres (≥0)*<br>*0 si studio*
3.7. Aviez-vous accès à un jardin ou une *kour* (espace privé) ?	☐ Oui ☐ Non
3.8. Aviez-vous un balcon, une terrasse/véranda ?	☐ Oui ☐ Non
3.9. Aviez-vous accès à un parc, jardin commun ou aire de jeux à proximité immédiate du logement (espace public) ?	☐ Oui ☐ Non
3.10. Aviez-vous accès à internet ?<br><br>**Si Oui**, par quels moyens :<br><br>- Ordinateur<br>- Tablette<br>- Smartphone<br>- TV<br><br>**Si Oui**, avez-vous eu des problèmes de connexion importants et fréquents ?	☐ Oui ☐ Non<br><br><br><br>☐ Oui ☐ Non<br>☐ Oui ☐ Non<br>☐ Oui ☐ Non<br>☐ Oui ☐ Non<br><br>☐ Oui ☐ Non

4) Exposition à l'épidémie

4.1. Avez-vous été directement touché(e) par le Covid-19 ?	☐ Oui j'ai présenté les signes<br>☐ Oui le test était positif<br>☐ Non<br>☐ NSP
4.2. Est-ce qu'un de vos proches a été touché par cette maladie (à la Réunion ou ailleurs) ?<br><br>**Si Oui**, est-ce qu'un de vos proches a été hospitalisé en raison du COVID-19 (à la Réunion ou ailleurs) ?	☐ Oui ☐ Non ☐ NSP<br><br>☐ Oui ☐ Non ☐ NSP

5) Etat psychologique

5.1. Actuellement, vous sentez-vous serein dans votre vie quotidienne ?	☐ Oui ☐ Non ☐ NSP
5.2. Souffriez-vous d'un trouble psychologique avant l'épidémie de COVID-19 ?<br><br>**Si Oui**, pensez-vous que le confinement a augmenté ce trouble psychologique ?	☐ Oui ☐ Non ☐ NSP<br><br>☐ Oui ☐ Non ☐ Sans réponse
5.3. Pensez-vous que l'épidémie et le confinement ont déclenché chez vous un trouble psychologique ?	☐ Oui ☐ Non ☐ Sans réponse
5.4. En ce moment, comment percevez-vous vos relations avec les autres ?	☐ Très mauvaises<br>☐ Plutôt mauvaises<br>☐ Plutôt bonnes<br>☐ Très bonnes
5.5. Avez-vous des inquiétudes par rapport à votre emploi ou à votre situation professionnelle dans l'avenir immédiat ?	☐ Oui ☐ Non ☐ Sans objet

5.6. Sur une échelle allant de 0 (absence totale de stress) à 10 (stress maximal imaginable) quel est votre niveau de stress **depuis le début de la période de confinement** ?	☐ 0<br>☐ 1<br>☐ 2<br>☐ 3<br>☐ 4<br>☐ 5<br>☐ 6<br>☐ 7<br>☐ 8<br>☐ 9<br>☐ 10
5.7. Quel est votre degré d'inquiétude face à la possibilité d'avoir le COVID-19 ? (De 1 (pas du tout inquiet) à 10 (très inquiet))	☐ 1<br>☐ 2<br>☐ 3<br>☐ 4<br>☐ 5<br>☐ 6<br>☐ 7<br>☐ 8<br>☐ 9<br>☐ 10
5.8. Avez-vous apprécié le confinement ?	☐ Oui ☐ Non ☐ Pas d'avis tranché<br><br>*Si réponse différente de Oui, aller au chapitre 6) Comportements addictifs p.13*

Si Oui, il vous a permis de :

5.9. Vous recentrer sur vous-même et votre famille ?	☐ Oui ☐ Non
5.10. Profiter du calme, de l'absence de bruit et d'embouteillages ?	☐ Oui ☐ Non
5.11. Respirer un air moins pollué ?	☐ Oui ☐ Non

5.12. Mettre en place des activités qui vous ont fait du bien comme :	
Des discussions	☐ Oui ☐ Non
De la lecture	☐ Oui ☐ Non
De l'écriture	☐ Oui ☐ Non
Voir des films, des séries	☐ Oui ☐ Non
Faire des jeux	☐ Oui ☐ Non
Cuisiner	☐ Oui ☐ Non
Bricoler	☐ Oui ☐ Non
Jardiner	☐ Oui ☐ Non
Ne rien faire	☐ Oui ☐ Non
Autre chose qui m'a fait du bien	☐ Oui ☐ Non

6) Comportements addictifs

Pendant la période du confinement, aviez-vous l'impression de :

6.1. Utiliser les écrans plus que d'habitude	☐ Oui ☐ Non ☐ N'utilise pas
6.2. Boire plus d'alcool que d'habitude ?	☐ Oui ☐ Non ☐ Ne boit pas
6.3. Fumer plus (tabac/vaporette-cigarette électronique) que d'habitude ?	☐ Oui ☐ Non ☐ Ne fume pas
6.4. Fumer plus (cannabis/zamal/chimique) que d'habitude ?	☐ Oui ☐ Non ☐ Ne fume pas
6.5. Avez-vous organisé des « apéros virtuels » sur les réseaux sociaux avec vos amis ?<br><br>**Si Oui**, combien de fois par semaine en moyenne :	☐ Oui ☐ Non<br><br>|__|__| *recueillir le nombre exact (≥1)*

7) Statut nutritionnel

7.1. Quel est votre poids actuel :	|__|__|__|kg ☐ NSP<br>*Arrondir à l'entier le plus proche si la réponse comporte une décimale*
7.2. Quelle est votre taille actuelle :	|__|__|__|cm ☐ NSP<br>*Arrondir à l'entier le plus proche si la réponse comporte une décimale*
7.3. Pendant la période de confinement, pensez-vous avoir :	☐ Pris du poids<br>☐ Perdu du poids<br>☐ Mon poids n'a pas changé<br>☐ NSP
7.4. Si prise de poids ou perte de poids, de combien de kilogrammes :	☐ Moins de 2 kg<br>☐ Entre 2 et 5 kg<br>☐ Plus de 5 kg<br>☐ NSP
7.5. Avant le confinement, pratiquiez-vous une activité sportive dans un club ou une association ?	☐ Oui ☐ Non
7.6. Avant le confinement, utilisiez-vous un équipement de quartier pour faire du sport (comme : terrain de foot, parcours de santé, etc.) ?	☐ Oui ☐ Non
7.7. Pendant la période de confinement, avez-vous l'impression que votre activité physique a été :	☐ Diminuée<br>☐ Augmentée<br>☐ N'a pas changé par rapport à d'habitude<br>☐ Sans objet (personne handicapée)

7.8. Pendant la période de confinement, avez-vous eu des difficultés à vous procurer certains aliments, comme :<br><br>- Fruits et légumes frais<br>- Ails/oignons<br>- Œufs<br>- Farine<br>- Autre<br>- Si autre, à préciser :	☐ Oui ☐ Non<br>☐ Oui ☐ Non<br>☐ Oui ☐ Non<br>☐ Oui ☐ Non<br>☐ Oui ☐ Non<br>____

8) Recours aux soins

8.1. Pendant le confinement, avez-vous dû reporter ou décaler au moins un rendez-vous médical ?<br><br>**Si Oui**, était-ce :<br><br>- De votre initiative<br>- A la demande du secrétariat du médecin<br>- Pour une autre raison	☐ Oui ☐ Non<br><br><br><br>☐ Oui ☐ Non<br>☐ Oui ☐ Non<br><br>☐ Oui ☐ Non
8.2. Pendant le confinement, avez-vous pu bénéficier de la téléconsultation ou de la télésurveillance ?	☐ Oui ☐ Non
8.3. Quelle est votre couverture maladie ?	☐ CMUc<br>☐ Mutuelle<br>☐ Pas de couverture complémentaire
8.4. Etes-vous atteint(e) d'une maladie chronique ou d'un problème de santé qui nécessite de consulter régulièrement le médecin ?<br><br>(Comme par exemple : diabète, hypertension artérielle, maladies du cœur, maladies des reins, cancers)	☐ Oui ☐ Non ☐NSP<br><br>*Si pas de maladies chroniques, aller au chapitre 9) Santé des enfants p.16*

8.5. Si vous êtes habituellement traité(e) pour votre maladie ou problème de santé, avez-vous rencontré un problème pour adapter / modifier votre traitement pendant le confinement ?	☐ Oui ☐ Non

9) Santé des enfants

9.1. Pendant le confinement, avez-vous remarqué ou entendu parler de violences faites aux enfants dans le voisinage ou votre entourage à la Réunion ?	☐ Oui ☐ Non
9.2. Avez-vous des enfants mineurs (âgés de moins de 18 ans) ?	☐ Oui ☐ Non<br><br>*Si pas d'enfant mineur, aller au chapitre 10) Violences en direction des femmes p.17*
9.3. Si Oui, ont-ils vécu le confinement avec vous sous le même toit ?	☐ Oui ☐ Non<br><br>*Si Non, aller au chapitre 10) Violences en direction des femmes p.17*

Si Oui, pendant le confinement :

9.4. Avez-vous ressenti qu'il(s) étai(ent) stressé(s), anxieux, ou qu'il(s) posai(ent) des questions montrant leur inquiétude ?	☐ Oui ☐ Non ☐ Non applicable (petit enfant)
9.5. Avai(en)t-il(s) des troubles du sommeil ?	☐ Oui ☐ Non ☐ Non applicable (petit enfant)
9.6. Avai(en)t-il(s) des problèmes de concentration ou d'attention ?	☐ Oui ☐ Non ☐ Non applicable (petit enfant)

9.7. Avai(en)t-il(s) des difficultés d'apprentissage pendant les enseignements scolaires à distance ?	☐ Oui ☐ Non ☐ Non applicable (petit enfant)
9.8. Avai(en)t-il(s) des troubles du comportement alimentaire ?	☐ Oui ☐ Non ☐ Non applicable (petit enfant)
9.9. Avai(en)t-il(s) accès à un objet connecté (ordinateur ou une tablette ou smartphone) ?	☐ Oui ☐ Non ☐ Non applicable (petit enfant)

Le volet suit ne concerne que les participantes. Pour les hommes, passer directement aux commentaires libres en fin de questionnaire. p.18

10) Violences en direction des femmes

Pouvez-vous me dire si vous avez été victime d'une ou plusieurs des 9 situations suivantes :

10.1. Utiliser la pandémie comme excuse pour augmenter le contrôle des finances de la famille	☐ Oui ☐ Non
10.2. Priver sa famille de nourriture, de médicaments, de gel hydroalcoolique	☐ Oui ☐ Non
10.3. Menacer ou empêcher la famille, y compris les enfants, de consulter un médecin en cas de symptômes ou cacher leur carte vitale	☐ Oui ☐ Non
10.4. Surveiller et critiquer sa partenaire	☐ Oui ☐ Non
10.5. Rendre sa partenaire responsable du comportement des enfants	☐ Oui ☐ Non

10.6. Isoler la famille, en surveillant ou empêchant les appels téléphoniques, les mails, l'accès aux réseaux sociaux en ligne, etc.	☐ Oui ☐ Non
10.7. Forcer les membres de sa famille à rester dans certains endroits de la maison (chambre, garage…)	☐ Oui ☐ Non
10.8. Retourner vivre au domicile de l'agresseur dont elle était séparée ou voir l'agresseur revenir au domicile occupé par la victime	☐ Oui ☐ Non
10.9. Utiliser la violence verbale ou physique en rendant le confinement responsable de son comportement	☐ Oui ☐ Non

Texte à lire :

« Par rapport aux situations rencontrées ci-dessus, si vous souhaitez être aidée et soutenue, vous pouvez contacter le Planning Familial de la Réunion au N° vert 0800 08 11 11 (choisir 974). »

Commentaire libre (en clair) :

____

____

____

« Merci pour votre participation à cette enquête. »
